# Entangling the Enemy: Ecological, Systematic, and Medical Implications of Dermestid Beetle Hastisetae

**DOI:** 10.3390/insects12050436

**Published:** 2021-05-12

**Authors:** Enrico Ruzzier, Marcin Kadej, Andrea Di Giulio, Andrea Battisti

**Affiliations:** 1 Department of Agronomy, Food, Natural Resources, Animals and the Environment (DAFNAE), University of Padova, Viale dell’Università 16, Legnaro, I-35020 Padova, Italy; andrea.battisti@unipd.it; 2 Department of Invertebrate Biology, Evolution and Conservation, University of Wrocław, POL-51148 Wrocław, Poland; marcin.kadej@uwr.edu.pl; 3 Department of Sciences, University of Roma Tre, I-00146 Rome, Italy; andrea.digiulio@uniroma3.it

**Keywords:** defensive behavior, larva, morphology, skin beetles, SEM

## Abstract

**Simple Summary:**

Larvae of Dermestidae Megatominae possess modified setae, called hastisetae, that serve as the primary defense against predators. These setae, beside a possible usage as discriminant character in larvae identification, are important contaminants of stored products, work and living environments. Exposure to hastisetae causes allergic reactions in humans and the insurgence of skin rashes, asthma, conjunctivitis, and digestive system inflammation. Very little is known about their mechanism of action, because the mechanical trap and their capability to penetrate throughout vertebrate epithelia remain unclear. The primary aim of the present contribution is to increase the general knowledge about hastisetae, exploring their diversity and fine morphology among genera and species. The insertion on integument, the pedicel, the shaft, and the apical head are illustrated in detail, and the first observation of active defensive behavior based on hastisetae is recorded and presented. The internal structure and the apex of the hastisetae are characterized for the first time, offering new interpretations in the action of hastisetae against vertebrates. Furthermore, possible implications to the systematics of skin beetles are proposed based on the results of the study.

**Abstract:**

Hastisetae are modified setae typical of Dermestidae Megatominae and are a primary defensive tool of both larvae and pupae against invertebrates and possibly vertebrates. Given their unique morphological features, hastisetae have recently been suggested as an additional character useful for larvae identification and possible source of information to clarify the systematics of Megatominae. Hastisetae are also recognized as important contaminants of stored products, work and living environment; in particular, the exposure to hastisetae seems to cause allergic reactions and the insurgence of skin rashes, asthma, conjunctivitis, and digestive system inflammation in humans. Starting from these basic concepts, the present paper provides a detailed description of the hastisetae of some Megatominae. Fine morphology of external and internal microstructures of the hastisetae is shown and compared at the genus level. The insertion on integument, the pedicel, the shaft, and the apical head are illustrated in detail, and the first observations of active defensive behavior based on hastisetae are recorded and presented. Possible implications to the systematics of skin beetles are proposed based on the results of the study.

## 1. Introduction

Among terrestrial arthropods, only two groups have evolved specialized setae intended to entangle predators: Myriapoda: Polyxenidae [1] and Coleoptera: Dermestidae, Megatominae Leach, 1815. Those setae are generally referred to as hastisetae or spear-like setae (Latin, *hasta*). Hastisetae are present only on larval tergites: on the thoracic segments they are generally scattered and in low numbers in respect to the other parts of the body. Abdominal tergites present a wider distribution pattern, from hastisetae covering the major parts of the tergal disc up to proper setae fields located at the posterior corners of tergites [2]. Aside from the hastisetae, the tergites present thorny setae, called spicisetae.

Hastisetae of Megatominae larvae constitute a mechanical trap, entangling and possibly leading predators to death [2]. Hastisetae, single or aggregated, are an efficient defensive mechanism aimed to entangle hairs and body appendages of invertebrates such as antennae, legs, and mouthparts. Nutting and Spangler [3] were the first to analyze the defensive function of hastisetae against invertebrates and vertebrates. They systematically fed, in artificial conditions, different groups of arthropods such as ants, beetles, earwigs, mantids, bugs and spiders, and then amphibians (toads), reptiles (geckos and skinks), birds and mammals (mice and squirrels) with larvae of the Megatominae beetle *Trogoderma* Dejan, 1821. Although all conditions were coercive and could hardly happen in the natural environment, they proved that the hastisetae discourage, entangle, or lead arthropod predators to death. A particular interest was the discouraging effect that these setae caused in arthropods that do not commonly prey on *Trogoderma* larvae and on toads. Reptiles, birds, and mammals apparently did not show any specific response after the exposition to *Trogoderma*, with the exception of geckos, that apparently may have died of intestinal blockage caused by hastisetae [3].

In 1976, Mills and Partida [4], and subsequently Kokubu and Mills [5], observed the entanglement mechanism of hastisetae, identified in the head of the hastisetae, the main structure involved in the defense mechanism. Although the hastisetae head is well illustrated in both papers, the true mechanism of action of this structure is only briefly discussed. It is thanks to Elbert [6] and Elbert [7] that a little more is known about the general structure and ultrastructure of hastisetae, and we are now capable of linking morphology to function. Elbert firstly provided an overview of hastisetae, comparing different species, and focused his attention on the head of hastisetae [6] and setal insertion on the larval integument [7]. He discovered that hastisetae are hollow along their entire length and that they seem to possess an opening at the apex [6,7]. Furthermore, the author observed how the shape and microsculpture of the seta head varies among species and genera [6] and provided the first and only investigation of hastisetae insertion [7]. Hastisetae are also recognized as important contaminants of stored products, work and living environments, because several dermestid beetles are synanthropic [8]. Particularly, hastisetae seem to be involved in allergic reactions through skin contact, ingestion, or inhalation, and causing symptoms such as skin rashes, asthma, conjunctivitis, and digestive system inflammation [9,10].

Even though hastisetae and their evolution is considered one of the key factors of the biological success of Megatominae [11,12,13], the most species-rich subfamily among Dermestidae [14] which constitute a non-negligible threat to human health [15], their structure and mechanism of action remain largely unresolved and restricted to a few cases, as summarized in Ruzzier et al. [2]. The purpose of this contribution is to explore hastisetae diversity, providing more information about their morphology and to investigate their possible usage in undertaking systematic and evolution of Megatominae; in particular, the research aimed to characterize the fine morphology of hastiseta, identifying those traits useful for its function against other invertebrates and to clarify if and how these setae may affect humans. Furthermore, knowing their important roles as contaminants and pests, this research focused its attention on selected genera to identify possible morphological traits useful for their identification using hastisetae.

## 2. Materials and Methods

### 2.1. Study Organism

Megatominae is the most species-rich subfamily among Dermestidae, totaling more than 800 described species worldwide [14]. This group is characterized by having short-living, often aphagous adults, and long-living, highly specialized larvae [13]. Megatominae larvae feed on various substrates, usually scarce in water content and poor in nutrients; as a consequence, their larval development is considerably slower in comparison to other dermestids. The capacity to feed on substrates unusable to most other invertebrates has meant that Megatominae have guaranteed themselves an ecological niche practically devoid of competitors. Due to their plasticity in exploiting trophic resources of both animal and plant origin and their long persistence, several Megatominae are primary pests and contaminants of stored goods, clothes, or artifacts with various purposes [16]. Furthermore, a discrete number of species are synanthropic and have been diffused by human means, becoming cosmopolitan in some cases. However, the important metabolic investment necessary for such a food source has important repercussions on larval development, determining a lengthening of the cycle and prolonged persistence of the larva in the environment.

The evolution of hastisetae as a primary defensive structure against threat is considered one of the key events that ensures the success of Megatominae [2], especially with a view to optimize energy investment in larvae. Hastisetae are present throughout the larval development and regenerate with each molt. The exuvia contains the hastisetae; therefore, their persistence creates an environment that provides a buffer—a discourage zone—against predators. Furthermore, Megatominae larvae pupate within their own exuviae, benefiting from hastisetae for pupa protection. This strategy is so efficient that Megatominae pupae lack gin-traps, defensive structures present on the abdominal segments of other dermestids deprived of hastisetae [11,12,13]. The presence and long persistence of hastisetae on the environment, both free and attached to the exuviae, and on the larvae, makes them important contaminants that can raise health issues for humans.

### 2.2. Examined Materials

The genera considered were *Anthrenocerus* Arrow, 1915, *Anthrenus* Geoffroy, 1762, *Ctesias* Stephens, 1830, *Thaumaglossa* Redtenbacher, 1867, *Megatoma* Herbst, 1791 and *Trogoderma* Dejan, 1821, for a total of 19 taxa (Table 1). Megatominae larvae are generally difficult to collect; therefore, it was complex to obtain larvae in number, especially identified to a species level. The majority of the materials used here belonged to the collection of Marcin Kadej (University of Wrocław, Poland) and have been identified by the author. Those taxa with live larvae that were available at the time of this study (*Anthrenus* spp. 1, *Anthrenus* spp. 2, *Ctesias serra* (Fabricius, 1792), *Megatoma undata* (Linnaeus, 1758), *Trogoderma* spp. 1, *Trogoderma* spp. 3) were kept in ventilated containers with dead crickets and mixed cereal flakes for food. Rearing containers were maintained at room temperature (20–22 °C). If larvae were not available, exuviae were used for the analysis.

### 2.3. Microscopy

Both larvae and exuviae were mounted on scanning electron microscopy (SEM) pin stubs, fixed using SEM adhesive carbon tabs, and golden-coated using a plasma sputter coater. Specimens were observed using a JSM Jeol 6490 SEM (CEASC, Università degli Studi di Padova, Padova, Italy). Ion milling, intended to dissect hastisetae, was performed using a Dualbeam FIB/SEM mod. Helios Nanolab 600 FEI Company (LIME, Università degli Studi Roma Tre, Rome, Italy); this apparatus is capable of selectively ablating (milling process) a previously marked region of the sample by using a focused ion current from a gallium source. The milling process can be interrupted every few nanometers to take high-resolution images of the cross sections with the SEM column. To observe the inner structure of the hastiseta with transmitted light, hastisetae of *M. undata* and *Anthrenus* spp. were mounted on microscope slides using Euparal mounting medium and photographed using an Optikam B10 Digital Camera mounted on a Askania RML microscope.

### 2.4. Defensive Behaviors Observation-Experiment

To preliminarily record if different genera displayed distinct defensive behaviors and if these were in some way related to the morphology of the hastisetae, some behavioral trials were conducted in a controlled environment. Five living larvae of *Trogoderma granarium* and ten of *Anthrenus* spp. were individually placed in separate Petri dishes. Under normal laboratory conditions and at room temperature, larvae of each species were systematically stimulated on the body and on the caudal apex with an entomological micropin and the was response observed.

## 3. Results

**Figure 1:**
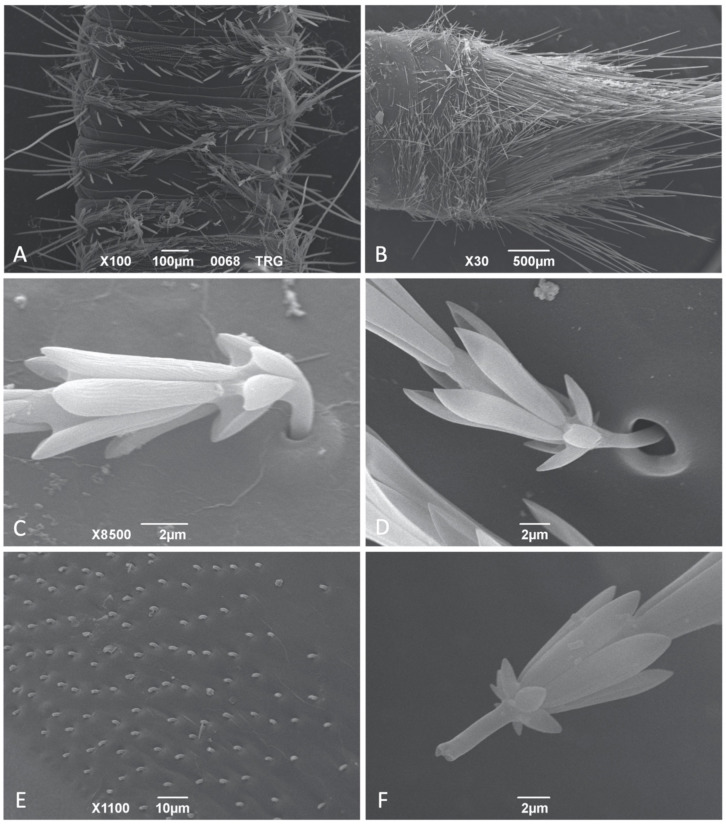
SEM photos of Megatominae larvae: (**A**) Dorsal view of the abdominal segments of *Trogoderma* spp. 3; (**B**) Dorsal view of the last abdominal segments of *Ctesias serra*; (**C**) *Trogoderma versicolor*, detail of the pedicel and insertion on the tergite; (**D**) *Ctesias serra*, detail of the insertion of the hastiseta on the tergite; (**E**) *Trogoderma* spp. 2, series of broken pedicels remaining after the detachment of one tuft of hastisetae; (**F**) *Ctesias serra*, detail of one detached hastiseta showing the breaking point on the pedicel.

Hastisetae are present in both thoracic and abdominal tergites of all larvae of the studied species, with their length and density varying substantially among genera, species and body segments; on the thoracic and abdominal tergites, with the exception of the caudal tufts, the hastisetae are aggregated in tufts or distributed on bands, as already shown in Beal [17,18,19], Peacock [20], Kiselyova [12,21], Kadej and Jaroszewicz [22], Kadej et al. [23,24,25], Kadej and Guziak [26], and Kadej [27,28,29,30,31]. The quantification of the hastisetae and the relative density is complex to assess, however even at a quick glance it is possible to observe that in all species, the caudal tufts are always the richest in bristles. *Anthrenocerus*, *Megatoma*, *Thaumaglossa*, *Trogoderma* have a relatively uniform vestiture of hastisetae along their entire body, and there is only a minimum difference in length between the hastisetae of the thorax and the first segments with those on the last segments of the abdomen (Figure 1A). On the contrary, *Anthrenus* and *Ctesias* possess short hastisetae on the thoracic and first abdominal segments, while they present long hastisetae creating tufts on the posterior margins of the fourth/fifth to seventh abdominal segments (Figure 1B).

### 3.1. Hastiseta Morphology

The morphology of the hastiseta was highly conserved among all the genera considered in this study and three main regions could be identified: pedicel, shaft, and the apical head [6,7].

#### 3.1.1. Hastiseta Insertion and the Pedicel

The hastisetae on the thorax and first abdominal segments are located on the sclerotized parts of the tergite. Setal tufts are inserted on the lateral sides of the tergites, with the hastisetae partially recumbent and oriented from the side to the center of the tergite. The pedicel, the most basal part of the hastiseta responsible for connecting it with the integument, has cylindrical section and does not present any surface roughness or sculpture (Figure 1C). The pedicel inserts almost directly on the cuticle, as could be observed in *Thaumaglossa* and *Trogoderma* (Figure 1C), or present a larger socket, usually in the form of a papilla, as in *Anthrenus* and *Ctesias* (Figure 1D). Preliminary observations allowed observations of the chitinous sheath inside the socket, surrounding the pedicel (S10-F). The pedicel is remarkably short in comparison to the rest of the seta and its longitudinal axis is not aligned with that of the hastiseta (Figure 1C). As a consequence, the two parts are connected at an obtuse angle, and its amplitude can vary. It is this particular type of junction which ensures that the hastisetae remain relatively recumbent on the surface of the body, all oriented in the same direction. The orientation of the pedicels and the relative density with which the hastisetae are inserted into the tergites are visible when the hastisetae are removed (Figure 1E). It is particularly interesting to note that if the hastisetae are artificially removed or the larva has lost them, they break at the level of the pedicel, thus suggesting its role as a breaking point to favor the detachment of the hastisetae. In all cases studied, the break point on the pedicel was clear, well demarcated (Figure 1F), and in a few cases occurred close to the first rosette.

**Figure 2:**
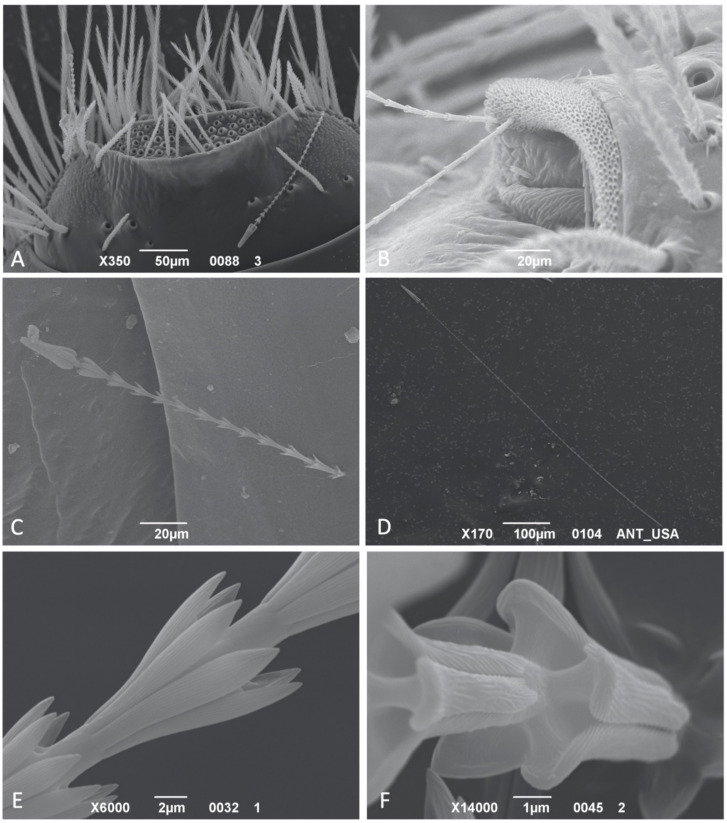
SEM photos of Megatominae larvae: (**A**) *Trogoderma* spp. 3, dorsal view of the 8th abdominal tergite showing the two patches of detached hastisetae at its corners; (**B**) *Anthrenus* (*Florilinus*) *olgae*, detail of the membranous part of the tergite bearing the insertion of the hastisetae; (**C**) Hastiseta of *Trogoderma granarium*; (**D**) Hastiseta from the caudal tufts of *Anthrenus* spp. 1; (**E**) *Megatoma undata*, lateral view of the rosettes; (**F**) *Trogoderma* spp. 3, anterolateral view of the rosette showing the chitin bridges supporting the scales.

In the last abdominal tergites (fourth/fifth to seventh), hastisetae are externally oriented, not recumbent, due to the short, straight pedicel, and higher in number compared to those on the previous segments. *Anthrenocerus*, *Megatoma*, *Trogoderma* present patches of hastisetae (tufts) inserted on the sclerotized part of the tergite (Figure 2A), while *Anthrenus*, *Ctesias* and *Thaumaglossa* possess hastisetae (tufts) inserted on the membranous integument at the posterior corners of the terga (Figure 2B). The number of such aggregations on tergites vary between genera. Contrary to what has been observed for the other genera, in which the insertion on the integument and the shape of the pedicel are uniform for all the hastisetae, those of *Anthrenus* and *Ctesias* showed unique features. *Anthrenus* possesses dense, circular sockets, close to each other, and apparently distributed without any peculiar pattern. Each socket has a diameter of approximately 2.5 μm and is characterized by having a well-developed collar that is raised above the surface of the integument (S2-B; S5-E). This collar partially wraps the first rosette of the hastiseta, so that the pedicel is not visible (S3-J). *Ctesias* presents insertions in the form of sockets roughly organized into rows, visible in the support file S10-H (https://zenodo.org/record/4700273#.YJzBl6gzZPZ) and partially illustrated in Kadej [30]. The distance between sockets belonging to the same row increases from ~2.5 μm, in the proximity of the spicisetae, up to ~10 μm, in the proximity of the posterior margin of the tergite. The sockets, ellipsoid in shape and with an approximated maximum width of 2.5 μm, present a slightly elevated and bordered collar. The pedicel inserts into a circular hole surrounded by the chitin sheath (S10-E, F). In both *Anthrenus* and *Ctesias*, the pedicel of the hastisetae inserted on the membranous part of the tergites is almost straight and aligned with the longitudinal axis of the hastiseta (Figure 1F; S9-M).

#### 3.1.2. Hastiseta Insertion and the Pedicel

The shaft dimensionally constitutes the main part of the hastiseta, varying in size from a few tens of microns, as in the thoracic hastisetae of *Trogoderma* (Figure 2C), to several hundred microns, observable in the caudal hastisetae of *Anthrenus* and *Ctesias* (Figure 2D). The constitutive unit of the shaft is the rosette, a set of scaly processes radially disposed around the hastiseta longitudinal axis and oriented externally and towards the head of the hastiseta (Figure 2C). The architecture of the rosettes was generally maintained in all the dermestids investigated, with scales varying among genera and species between five or seven, rarely nine; the only exception was observed in some *Anthrenus* in which the basal rosettes of the hastisetae on the last abdominal segments were columnar (S8-L). The length of the rosettes is not uniform and varies, even among the rosettes of the same hastiseta. Each scale is connected to the main body of the shaft through one cuticle bridge for almost its entire length (Figure 2D). This bridge is short or almost inexistent, close to the proximal part of the rosette, while it is well developed in the distal part, so that the apex of the scale is always diverging to the central axis of the hastiseta. Each scale is lanceolate, with a distal apex that can be more or less sharp, and the lateral margins slightly bent over; the profile of the scales and their length varies among genera (S2-E; S13-E), and these differences are generally difficult to appreciate and quantify at species level. The dorsal part of the scales is covered in dense longitudinal knurls for all their length (Figure 2E), while the ventral side, when visible, consists of an amorphous matrix of chitin (S11-E).

**Figure 3:**
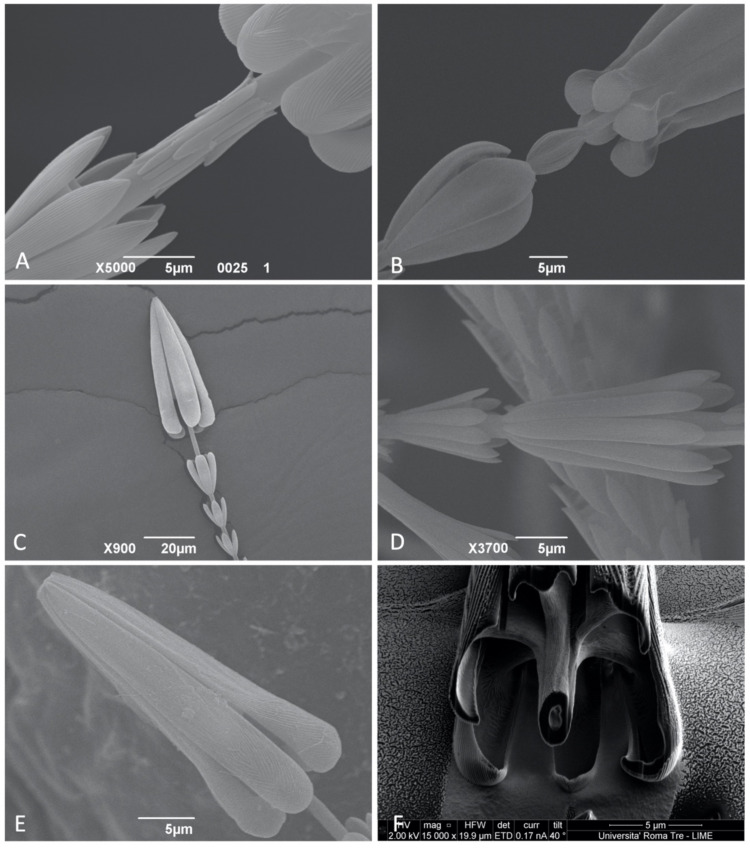
SEM photos of Megatominae larvae: (**A**) *Megatoma undata*, detail the hastiseta showing the irregular scales on the shaft; (**B**) *Ctesias serra*, detail of the last rosette and the bud-like modification of the shaft; (**C**) *Anthrenus* (*Anthrenus*) *latefasciatus*, hastiseta from the caudal tufts, detail of the apical part; (**D**) *Thaumaglossa rufocapillata*, size and shape comparison between the penultimate and ultimate rosette; (**E**) *Anthrenus* (*Florilinus*) *olgae*; head of hastiseta; (**F**) Diagonal section of the basal part of the head of the hastiseta of *Trogoderma variabile*.

The structure, shape, and size of the last rosette and the part of the shaft before the apical head were particularly interesting. Based on these features, the taxa studied can be subdivided in four morphological groups: *Trogoderma*, *Anthrenocerus* and *Megatoma* are characterized by having ultimate rosette of similar size or only slightly larger than the previous. In addition, the three genera possess a series of irregular scales on the shaft, just before the apical head. These recumbent scales are concentrated in a single point and arranged in a ring around the shaft. Each of these scales present the same knurls as rosettes (Figure 3A). *Ctesias* possesses a tulip-shaped ultimate rosette, substantially larger in size than the previous (Figure 3B). Unlike the previous two, this genus presents an enlarged shaft between the rosette and the apical head; the bulge is covered with irregular scales and resembles a bud (S10-O). *Anthrenus* presents an ultimate rosette larger but almost identical in shape to the previous rosettes (S2-C; S4-A). A peculiarity of this genus is the absence of scales or bulges on the shaft between the rosette and the apical head of the hastiseta (Figure 3C,E). *Thaumaglossa* possesses a unique combination of features, with the ultimate rosette significantly larger than the previous (Figure 3D). This rosette has a more compact appearance and is constituted by nine scales, unlike the previous, which has seven. Moreover, this taxon presents the same ring of irregular scales on the shaft as in *Trogoderma*, *Anthrenocerus* and *Megatoma*.

#### 3.1.3. Apical Head

The apical head is a truncated-cone, anchor-like structure constituted by a series of long processes posteriorly oriented with respect to the apex of the hastiseta; the number of processes varies in accordance with the number of scales on the last rosette. All the processes are identical and organized radially respect the longitudinal axis of the hastiseta (Figure 3E). Each process is constituted by one linear part starting on the apex of the head and ending distally with a crook/hook; this last part is usually convex on the outside and concave on the inside (Figure 3F). The process is connected to the shaft in its basal part throughout a chitin bridge similar to that of rosettes (Figure 3F; S20). This particular arrangement causes the processes of the hastisetal head, and in particular the distal hook, to be oriented against the last rosette. All the processes are covered for their entire length in knurls always antero-laterally oriented with respect to the apex of the hastiseta.

**Figure 4:**
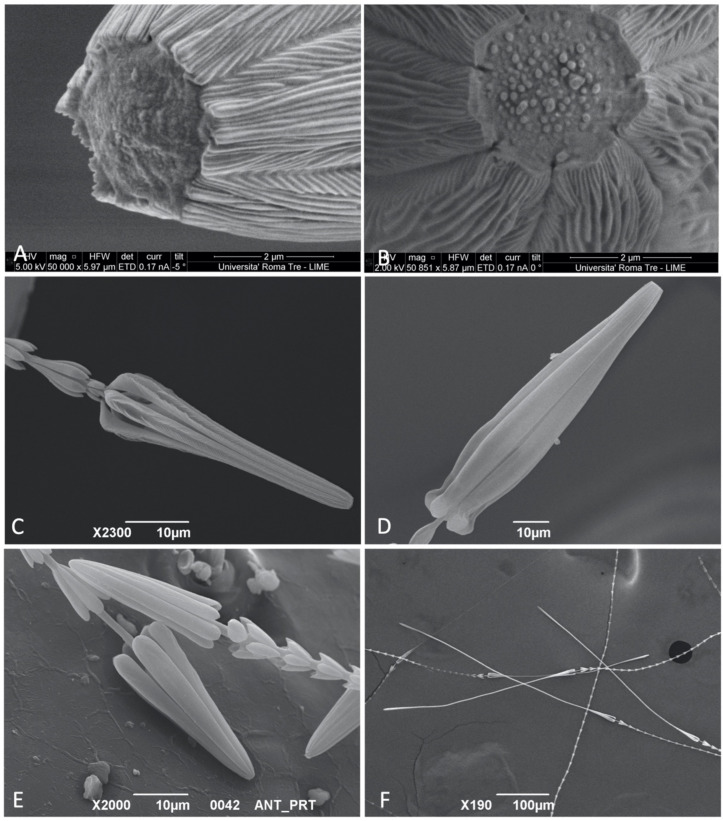
SEM photos of Megatominae larvae: (**A**) Detail of the blunt apex and the circular depression of *Trogoderma variabile*; (**B**) Frontal view of the circular depression on the apex of the hastiseta showing the amorphous matrix inside it; (**C**) Head of the hastiseta of *Trogoderma* sp. 2; (**D**) Head of the hastiseta of *Ctesias serra*; (**E**) Head of the hastiseta of *Anthrenus* sp. 2; (**F**) Head of the hastiseta of *Anthrenus picturatus makolskii*.

A common feature of the hastisetae is the blunt apex of the seta (Figure 4A); in all cases studied, at the convergence (base/origin) of the processes and exactly in correspondence to the terminal part of the shaft, the hastiseta presented one circular depression (2.5–3.0 μm in diameter). The margins of this depression can be more or less sharp and irregular depending on the incidence angle of the processes. It was impossible to define any further feature related to the circular depression, because in both larvae and exuviae it was filled with an amorphous matrix of unclear origin (Figure 4B). As for the last rosette, the head of the hastiseta presents a substantial degree of variability among genera and species. Following this variability, the studied taxa can be separated in two main morphogroups: *Anthrenocerus*, *Ctesias*, *Megatoma*, *Thaumaglossa* and *Trogoderma* are all united by having the head of the hastisetae of a relatively homogeneous shape and size on all body segments. *Anthrenus* instead presents hastisetae on the tufts on the fifth to seventh, segments in which the head substantially differs from those of the remaining abdominal and thoracic terga.

In *Trogoderma* and *Anthrenocerus*, the head of the hastiseta has a similar shape and size between tergites and caudal hastisetae. One synapomorphy of the two genera is the presence of one longitudinal depression along each process of the head of the hastiseta (Figure 4C). This depression, which can vary among species in length and depth, is defined by the convergence of the transversal or by the presence of few longitudinal ridges. In *Ctesias*, the head of the hastiseta is identical in both thoracic and caudal hastisetae and it is characterized by a unique profile, which is umbrella-shaped (Figure 4D). This part of the hastiseta possesses a flared profile, with a bulge roughly near the distal second third of its length, and then strongly narrowed in the distal fourth. The apical bending of each longitudinal process is particularly globular, also due to the strong narrowing that precedes it. Each process of the head of the hastiseta is densely covered in fine ridges, clearly observable only at magnification >3500×. The genus *Megatoma* possesses a head of the hastiseta that, for its proportion and in respect to the rosettes and general shape (truncated cone), resembles those of *Trogoderma* and *Anthrenocerus*. However, compared to the latter, the longitudinal processes do not present any type of depression or notch. *Thaumaglossa* has homogeneous hastisetae on the whole body; however, as for the last rosette, the number of longitudinal processes that constitute the head of the hastiseta is nine, not five or seven as in the other genera. *Anthrenus* presents the most complex set of hastisetal heads compared to other genera, and some of these features have been suggested as one of the possible larval diagnostic characters in Kadej et al. [23,24] and Kadej [31]. *Anthrenus* is so far the only genus that possess two different types of head of hastiseta, one on the shortest thoracic and first abdominal segment hastisetae and a different type on the longer caudal tufts; these heads differ substantially in size or shape, with the exception of *Anthrenus* (*s*. *str*.) *latefasciatus* and *Anthrenus* spp. 2 (Portugal) in which the heads of the hastisetae are both conical and almost identical in size (Figure 4E). *Anthrenus* (*F*.) *olgae* and *Anthrenus* sp. (USA) are characterized by having the head of the hastisetae on the last abdominal segments conical and about 1.7 times longer than those on the previous, while those of *Anthrenus* (*H*.) *fuscus*, *Anthrenus* (*H*.) *polonicus* are 2.0× and 1.3× longer, respectively. *Anthrenus* (*s*. *str*.) *scrophulariae* and *Anthrenus* (*s*. *str*.) *picturatus makolskii* are the only species that possess oblong hastisetae heads on both thoracic and abdominal hastisetae. In both species, the longitudinal processes are relatively close to each other and not divergent from the central axis of the hastiseta. *Anthrenus* (*s*. *str*.) *scrophulariae* presents oblong–conical heads on the hastisetae of the thorax and first thoracic segments, while on the last tergites, the head is bottle-shaped, 3.7 times longer than those on thorax. *Anthrenus* (*s*. *str*.) *picturatus makolskii*, on the other hand, possesses hastisetae with an oblong, parallelepiped head on thorax; at the level of the abdominal tergites, with the exclusion of the long tufts of hastisetae, these setae are mixed with hastisetae having a more elongated head (Figure 4F). A peculiarity of this species is the hastisetae constituting the tufts on segments 5–7, in which the head of the hastiseta is remarkably stretched, about 7.7 times the length of those on the other tergites. This peculiar type of head is slightly enlarged in the proximity of the apex of the longitudinal processes, and it displays a light-clockwise torsion along its longitudinal axis.

**Figure 5:**
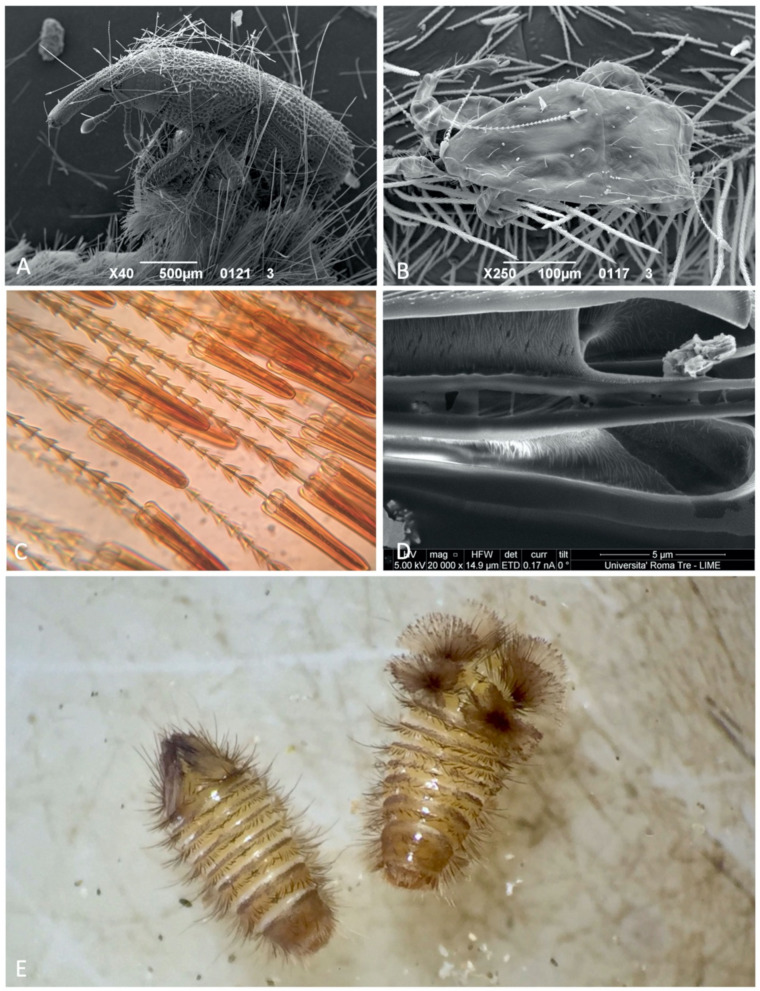
(**A**) SEM photo of a *Sitophilus* spp. (Curculionidae) trapped on the exuvia of *Megatoma undata*; (**B**) SEM photo of a Mesostigmata mite entangled by hastisetae on a larva of *Trogoderma* spp. 1; (**C**) Hastisetae of *Megatoma undata* mounted in Euparal showing the darker, empty core; (**D**) SEM photo of a section of the head of the hastisetae of *Trogoderma variabile* illustrating the internal cavity of the stalk filled with the amorphous matrix; (**E**) Larvae of *Anthrenus* spp.: left specimen in a relaxed condition and hastisetae at rest; right specimen displaying defensive behavior with raised hastisetae.

On two different occasions during the preparation of the specimens for the SEM, two different groups of arthropods were observed while trapped by hastisetae. These two events, both natural expositions to hastisetae and not forced as in Nutting and Spangler [3] or Kokubu and Mills [5], confirmed once again the primary role of hastisetae as extremely efficient mechanical traps in entangling predators, parasitoids, and possibly competitors. The first case was regarded to some *Sitophilus* spp. (Coleoptera: Curculionidae) infesting the rearing substrate of *M. undata*. All specimens were found dead, possibly due to the effort to free themselves, with antennae, tarsi, and scales covered in hastisetae. What was particularly interesting was the fact that those beetles did not come into contact with live larvae (that were no longer present in the enclosure), but instead with loose hastisetae contaminating the substrate or larval exuviae. This condition suggests that hastisetae maintain their functionality over time, even if detached from the larval tergites. On one occasion, a *Sitophilus* remained totally stuck on a larval exuvia, providing direct proof of how synergic actions of multiple hastisetae constitute a trap almost impossible to escape (Figure 5A). In the second case, Mesostigmata mites were found entangled by *Trogoderma* hastisetae, with some of them still trapped on the dorsum of the larvae (Figure 5B). Even in this case, the trapped arthropods had tarsal claws and trichomes entangled with hastisetae but, differently from the previous case, the studied larvae were alive before the preparation for the SEM. It is plausible that these mites were predators or simply co-occurring species on the same substrate.

#### 3.1.4. Internal Structure

Both Euparal slides and ion milling revealed that in the genera considered, hastisetae are hollow, and the internal cavity extends homogeneously from the subcuticular structures up to the apex of the hastiseta (circular depression) (Figure 5C). Longitudinal sections of the hastiseta seem to indicate the presence inside the lumen of an amorphous substance of unclear origin (Figure 5D).

### 3.2. Response to Disturbance

*Trogoderma* and *Anthrenus* displayed two completely different responses to stimulation. *Trogoderma* did not show any substantial modification in body posture but stopped every activity after the stimulation in almost all trials conducted. If stimulated locally with the tip of the micropin, the larvae were capable of contracting body segments and orientating the tergites bearing hastisetae in the direction of the stimulus. Sporadically, in the case of persistent disturbance, the larva abandoned the passive defense and tried to escape. *Anthrenus* instead always demonstrated a defensive behavior of an aggressive type, which in some ways resembled that of a porcupine. At a minimum stimulus, the larvae stopped all activity, contracted the body segments, and markedly arched the last abdominal segments. This strategy determined that the tufts of hastisetae of the last abdominal tergites, usually recumbent, were raised and opened like a fan (Figure 5E); this defensive posture was held by the larva until the disturbance continued. The defensive response in *Anthrenus* was induced by both a direct stimulation on the body and a mechanical stimulation of the long tufts of supranal bristles [32].

## 4. Discussion

### 4.1. Morphology and Biomechanics of Hastisetae

The morphological data collected here are consistent with the observations provided in Elbert [7], in which the pedicel extends up to the deep layer of the endocuticle. This trait indicates that hastisetae must no longer be considered true setae but instead modified setae (Battisti et al. [33]); further support to the present interpretation is given by the existence of the breaking point that permits the detachment of the hastiseta. Observations confirmed that the head is the key structure in hooking and trapping body parts of the threatening organism that comes in contact with it. In both Mills and Partida [4] and Kokubu and Mills [5], and exactly as in the samples studied here, scales, claws, and hairs were systematically and irrecoverably trapped among the longitudinal processes of the head of the hastiseta. The unique radial symmetry, the septation, and the gaff shape of the apical part of the longitudinal processes make the head a perfect trap, capable of guaranteeing an anchorage that cannot be removed. The head is structured in such a way that whatever organism is trapped, the more it tries to free itself the more it is stuck. An arthropod which is trying to remove the hastiseta is inclined to pull; therefore, its body parts end up more and more trapped due to the knurls present on the longitudinal processes and the reduction in the lumen between adjacent longitudinal processes of the head of the hastiseta. It should also be considered that the effort in freeing by the arthropod determines the breaking of the pedicel and the detachment of the hastisetae from larval integuments. In this way, the detached hastisetae synergically amplify the entangling effect due to the friction and trapping caused by the rosette against other rosettes and body parts. The efficiency of trapping bristles and other structures is most likely determined by the combined action of the last rosette and the hastiseta head, in which the opposite orientation of the two parts would increase the trapping capacity of the hastiseta.

It is interesting to note that Megatominae apparently solved the issue of increasing the trapping efficiency of the hastiseta at least in two different ways: *Anthrenus* evolved a particularly enlarged ultimate rosette which apparently has the function of guiding the bristles and scales towards the head trap, while *Trogoderma*-like Megatominihas developed accessory scales on the shaft as alternative structures to achieve the same result. The capability of *Anthrenus* in rising hastisetae only when disturbed suggests a more efficient defensive strategy against threats, possibly contributing to the great success of the genus that constitutes approximately the 30% of all Megatominae. Long hastisetae are potentially more efficient in discouraging and entangling predators; however, given their size, they may be subject to involuntary detachment or to create an impediment to the larva. It is plausible that *Anthrenus* evolved hastisetae on soft teguments, able to fold down in brushes under relaxed conditions. This type of strategy is documented in *Cryptorhopalum* Guérin-Méneville, 1838 [12] and, even if not directly observed, may also occur in *Ctesias* and *Thaumaglossa*. On the contrary, *Anthrenocerus*, *Megatoma* and *Trogoderma* have developed a static defense that possibly bases its success on the quantity and density of hastisetae covering the larvae. The substantial difference in morphological traits and defensive behavior may, at least among the Megatominae investigated, be attributable to the different ecological niches and environments colonized, as suggested in Zhantiev [13].

Among all the peculiarities emerged in the investigation, the role and origin of the circular depression on the tip of the hastiseta remains unclear. The fact that this structure is always present in all the taxa investigated and those pictured by Elbert [6] and Elbert [7], seems to suggest its involvement in the function of the hastiseta. The presence of the amorphous matrix in the circular depression and inside the lumen of hastisetae may indicate some sort of secretion; however, whether the compound has a deterrent or adhesive effect remains a future aspect to be investigated.

### 4.2. Systematics

The set of data collected, combined with the published information concerning the larval morphology and the phylogeny of the group, enabled identification of some features potentially helpful to define the systematics of Megatominae. *Anthrenocerus*, *Megatoma*, and *Trogoderma* are all united by possessing hastisetae located on the sclerotized part of the tergites and the presence of irregular scales on the shaft, just before the head of the hastiseta. This set of features is consistent with the lineage identified by Kiselyova and McHung [12] on the base of larval morphological traits and in partial agreement with the molecular phylogeny provided by Castalanelli et al. [34]. The resemblance of the hastisetae of *Anthrenocerus* and *Trogoderma*, especially the presence of longitudinal depression on the processes of the head of the hastiseta, suggests a close relationship between the two genera, as already indicated by Kiselyova and McHung [12] and Castalanelli et al. [34].

Morphological apomorphies of *Anthrenus* (enlarged last rosette, lacking irregular scales on the shaft) indicate its belonging to a distinct lineage with respect to the aforementioned genera, as shown in Kiselyova and McHung [12]. *Anthrenus* apparently diverged during the early stages of Megatominae evolution [34] and evolved a unique set of morphological and ethological traits. *Ctesias* and *Thaumaglossa* (Tribe Megatomini) possess a set of characteristics that suggest an intermediate position in respect to *Anthrenus* (Tribe Anthrenini) and the *Anthrenocerus*–*Trogoderma* lineage (Tribe Megatomini). These two genera share characteristics with *Anthrenus* hastisetae, on the membranous part of last abdominal tergites and the irregular scales/modified shaft before the apical head. *Ctesias* has a unique shape of the ultimate rosette, the presence of bud-like shaft modifications (that we may consider a proto set of scales), and the peculiar profile of the head of the hastisetae suggest a closer relation to *Anthrenus*, with which it shares elongated hastisetae on the last abdominal segments. It is interesting to note that *Ctesias* shares some similarities with Cretaceous amber fossils described by Poinar and Poinar [35].

The unique enlarged and modified ultimate rosette of *Thaumaglossa* agrees with the phylogeny provided by Kiselyova and McHung [12], in which the genus cluster in a separate clade with respect to *Anthrenocerus*, *Megatoma* and *Trogoderma*. *Megatoma* may be considered closer to *Trogoderma*-like Megatomini (Kadej [29]), with which its shares hastisetae similar in length on the abdominal segments, a last rosette similar in shape and size to the previous and the irregular scales on the shaft; this placement agrees with the results indicated in Beal [19] and Kiselyova and McHugh [12].

## 5. Conclusions

Hastisetae are modified setae that are released in the environment only through the mechanical rupture of the pedicel. Our observation suggests that hastisetae not only trap different subphyla of Arthropoda, but they target a broad spectrum of predator/parasite sizes; this functional plasticity provides a highly effective defensive mechanism that implies minimal energy investment in its implementation. In addition, the peculiar shape of the hastisetae suggests that they did not evolve as an offensive defensive structure against vertebrates and the effects observed, including those on humans, are fortuitous. This interpretation is supported by the shape of the hastisetae in which the blunt apex of the head renders them unable to penetrate through sclerified epithelia, such as the skin, unlike what is present on some lepidoptera and tarantulas where detachable setae are sharp [33]. Inflammation observed in humans, often affecting the soft oro/rhino-pharyngeal epithelium and conjunctiva, are most probably caused by the mechanical friction of the seta on the epithelia. Furthermore, the insurgence of the symptoms may be induced by chitin, proteins, and other compounds associated with the integument, and the way in which the inflammation manifests can be highly subjective due to the different sensitivities that each individual has towards these molecules.

In this regard of applied entomology, hastisetae may constitute a substantial help in contaminant identification, especially in the case of fragmented dermestid residues. The available information so far permits the identification at genus level of some of the most common pests and synanthropic taxa. However, in order to be able to progress and develop an identification tool at species level, it will be necessary to substantially increase the number of species to be included in the analyses and to develop a standard protocol for their observation. In fact, the way in which the seta is prepared, and its conservation state, may affect the possibility of appreciating specific, relevant characters.

Furthermore, a wider set of morphological data, inclusive of a good representation of worldwide Megatominae and *Trinodes* Dejean, 1821 (Subfam. Trinodinae; a genus that possess proto-hastisetae), can surely help in clarifying and resolving the systematic and evolution of the subfamily. Preliminary data are encouraging, and the morphological traits associated with hastisetae are consistent with the phylogeny based on larval characteristics defined by Kiselyova and McHung [12] and the molecular phylogeny proposed by Castalanelli et al. [34]. The increase in genetic sequences available, together with new information on the biology and ethology of the larvae, may help in clarifying whether specific hastisetal traits represent evolutionary convergence due to similar larval niches or are the result of a common origin.

## Figures and Tables

**Table 1:** List of the taxa included in the study.

Species	Stage	Origin	Supp. File
*Anthrenocerus australis* (Hope, 1843)	Larva	Australia	S1
*Anthrenus* (*Anthrenus*) *latefasciatus* Reitter, 1892	Exuvia	Kazakhstan	S2
*Anthrenus* (*Anthrenus*) *picturatus makolskii* Mroczkowski, 1950	Larva (ex ovo)	Poland	S3
*Anthrenus* (*Anthrenus*) *scrophulariae scrophulariae* (Linnaeus, 1758)	Exuvia	Poland	S4
*Anthrenus* (*Florilinus*) *olgae* Kalík, 1946	Larva (ex ovo)	Poland	S5
*Anthrenus* (*Helocerus*) *fuscus* Olivier, 1789	Larva (ex ovo)	Poland	S6
*Anthrenus* (*Helocerus*) *polonicus* Mroczkowski, 1950	Larva (ex ovo)	Poland	S7
*Anthrenus* spp. 1	Larva	USA (California)	S8
*Anthrenus* spp. 2	Exuvia	Portugal	S9
*Ctesias serra* (Fabricius, 1792)	Larva	Poland	S10
*Megatoma* (*Megatoma*) *undata* (Linnaeus, 1758)	Exuvia	Poland	S11
*Thaumaglossa rufocapillata* Redtenbacher, 1867	Larva	China	S12
*Trogoderma granarium* Everts, 1898	Larva	Czech Republic	S13
*Trogoderma simplex* Jayne, 1882	Larva	USA (California)	S14
*Trogoderma variabile* Ballion, 1878	Larva	USA (California)	S15
*Trogoderma versicolor* (Creutzer, 1799)	Larva (ex ovo)	Poland	S16
*Trogoderma* spp. 1	Larva	Italy	S17
*Trogoderma* spp. 2	Larva	Romania	S18
*Trogoderma* spp. 3	Larva	Italy	S19

## Data Availability

All data are available online at https://zenodo.org/record/4700273#.YJzBl6gzZPZ.

## References

[1] Eisner T., Eisner M., Deyrup M. (1996). Millipede defense: Use of detachable bristles to entangle ants. Proc. Natl. Acad. Sci. USA.

[2] Ruzzier E., Kadej M., Battisti A. (2020). Occurrence, ecological function and medical importance of dermestid beetle hastisetae. PeerJ.

[3] Nutting W.L., Spangler H.G. (1969). The hastate setae of certain dermestid larvae: An entangling defense mechanism. Ann. Entomol. Soc. Am..

[4] Mills R.B., Partida G.J. (1976). Attachment mechanisms of *Trogoderma* hastisetae that make possible their defensive function. Ann. Entomol. Soc. Am..

[5] Kokubu H., Mills R.S. (1980). Susceptibility of thirteen stored product beetles to entanglement by *Trogoderma* hastisetae. J. Stored Prod. Res..

[6] Elbert A. (1976). Elektronenmikroskopische Untersuchungen der Pfeilhaare verschiedener Arten der Anthreninae (Col. Dermestidae). Anz. Schädlingskunde Pflanzenschutz Umweltschutz.

[7] Elbert A. (1978). Die Pfeilhaare der Megatominae (Col., Dermestidae): Ein Abwehrsystem. Anz. Schädlingskunde Pflanzenschutz Umweltschutz.

[8] Hinton H.E. (1945). A Monograph of the Beetles Associated with Stored Products.

[9] Gorgojo I.E., De Las Heras M., Pastor C., Cuesta Herranz J., Sanz Maroto A. (2015). Allergy to Dermestidae: A new indoor allergen? [Abstract AB105]. J. Allergy Clin. Immunol..

[10] MacArthur K.M., Richardson V., Novoa R.A., Stewart C.L., Rosenbach M. (2016). Carpet beetle dermatitis: A possibly under-recognized entity. Int. J. Dermatol..

[11] Zhantiev R.D. (2000). Classification and phylogeny of dermestids (Coleoptera, Dermestidae). Entomol. Rev..

[12] Kiselyova T., McHugh J.V. (2006). A phylogenetic study of Dermestidae (Coleoptera) based on larval morphology. Syst. Entomol..

[13] Zhantiev R.D. (2009). Ecology and classification of dermestid beetles (Coleoptera, Dermestidae) of the Palearctic fauna. Entomol. Rev..

[14] Háva J. (2015). World Catalogue of Insects. Volume 13. Dermestidae (Coleoptera).

[15] Mullen G., Durden I. (2009). Medical and Veterinary Entomology.

[16] Athanassiou C.G., Phillips T.W., Wakil W. (2019). Biology and control of the khapra beetle, *Trogoderma granarium*, a major quarantine threat to global food security. Annu. Rev. Entomol..

[17] Beal R.S. (1956). Synopsis of the economic species of *Trogoderma* occurring in the United States with description of a new species (Coleoptera: Dermestidae). Ann. Entomol. Soc. Am..

[18] Beal R.S. (1960). Descriptions, biology, and notes on the identification of some Trogoderma larvae (Coleoptera, Dermestidae).

[19] Beal R.S. (1967). A revisionary study of the North American dermestid beetles formerly included in the genus *Perimegatoma* (Coleoptera). Misc. Publ. Entomol. Am..

[20] Peacock E.R. (1993). Adults and Larvae of Hide, Larder and Carpet Beetles and Their Relatives (Coleoptera: Dermestidae) and of Derodontid Beetles (Coleoptera: Derodontidae). Handbooks for the Identification of British Insects.

[21] Kiselyova T. (2002). Description of the larval and pupal stages of *Cryptorhopalum triste* LeConte (Coleoptera: Dermestidae), with notes on biology and rearing. Coleopt. Bull..

[22] Kadej M., Jaroszewicz S. (2013). Detailed morphological description of the mature larva of *Globicornis corticalis* (Eichhoff, 1863) (Dermestidae: Megatominae) with comparisons to related species. Zootaxa.

[23] Kadej M., Jaroszewicz S., Tarnawski D. (2013). Morphology of mature larvae of three species of the genus *Anthrenus* (Dermestidae: Megatominae: Anthrenini) with comparisons to related species. Ann. Entomol. Soc. Am..

[24] Kadej M., Jaroszewicz S., Tarnawski D. (2013). Comparative morphology and biology of mature larvae in the genus *Anthrenus* (Dermestidae: Megatominae: Anthrenini) with comparisons to related species. Ann. Société Entomol. Fr. N.S. Int. J. Entomol..

[25] Kadej M., Guziak J., Marczak D. (2017). A detailed updated description of the morphology of the larva of *Reesa vespulae* (Milliron, 1939) (Dermestidae: Megatominae: Megatomini). FL Entomol..

[26] Kadej M., Guziak J. (2017). Description of the larva of *Globicornis emarginata* (Gyllenhal, 1808) (Dermestidae: Megatominae). Ann. Zool..

[27] Kadej M. (2012). Detailed morphological description of the mature larva of *Anthrenus latefasciatus* Reitter, 1892 (Dermestidae: Megatominae: Anthrenini) with comparisons to related species. Zootaxa.

[28] Kadej M. (2012). Detailed description of the morphology of the last instar larva of *Trogoderma megatomoides* Reitter, 1881 (Dermestidae: Megatominae: Megatomini) with comparison to related species. J. Kans. Entomol. Soc..

[29] Kadej M. (2017). Larva and pupa of *Megatoma* (*s*. *str*.) *undata* (Linnaeus, 1758) (Coleoptera: Dermestidae) with remarks on biology and economic importance. Zookeys.

[30] Kadej M. (2018). Larva and pupa of *Ctesias* (*s*. *str*.) *serra* (Fabricius, 1792) (Coleoptera: Dermestidae) with remarks on biology and economic importance, and larval comparison of co-occurring genera. Zookeys.

[31] Kadej M. (2018). Contribution to the knowledge of the immature stages of Dermestidae with special emphasis on the larval morphology of the genus *Anthrenus* Geoffroy, 1762 (Megatominae: Anthrenini). Polish Entomological Monographs.

[32] Ma M., Burkholder W.E., Carlson S.D. (1978). Supra-anal Organ: A Defensive Mechanism of the Furniture Carpet Beetle, *Anthrenus flavipes* (Coleoptera: Dermestidae). Ann. Entomol. Soc. Am..

[33] Battisti A., Holm G., Fagrell B., Larsson S. (2011). Urticating hairs in arthropods: Their nature and medical significance. Annu. Rev. Entomol..

[34] Castalanelli M., Baker A., Munyard K., Grimm M., Groth D. (2012). Molecular phylogeny supports the paraphyletic nature of the genus *Trogoderma* (Coleoptera: Dermestidae) collected in the Australasian ecozone. Bull. Entomol. Res..

[35] Poinar G.O., Poinar R. (2016). Ancient hastisetae of Cretaceous carrion beetles (Coleoptera: Dermestidae) in Myanmar amber. Arthropod Struct. Dev..

